# Final Thoughts, Microorganisms Special Issue on Microbial Hemoglobins

**DOI:** 10.3390/microorganisms10020379

**Published:** 2022-02-06

**Authors:** Benjamin C. Stark

**Affiliations:** Department of Biology, Illinois Institute of Technology, Chicago, IL 60616, USA; starkb@iit.edu

The year 2021 marked the thirty-fifth anniversary of the discovery of microbial hemoglobins by Dale Webster and his colleagues. The importance of this work lay in its showing that hemoglobins are a much more ancient family of proteins and are much more widely distributed among taxa than previously recognized, and that, while their functions all involve oxygen in one way or another, these functions are variable. From a historical perspective, this Special Issue begins with a retrospective from Professor Webster and his colleagues about the earliest work that led to the identification of the first-known microbial hemoglobin (“VHb”, from the bacterium *Vitreoscilla*), as well as early studies on its metabolic roles and its use in practical applications to enhance industrial and environmental remediation processes.

Taymaz-Nikerei and Lara contribute to the discussion of the involvement of *Vitreoscilla* hemoglobin in intermediary metabolism, specifically addressing the “overflow effect,” which results from an excess of carbon supply over available oxygen. Using modeling of *E. coli* metabolism, they suggest VHb’s ability to mitigate this affect by increasing the oxygen supply to cells. Yu et al. contribute a detailed and comprehensive review of the current knowledge on the structure, biochemical properties, functions, and effects on metabolism of VHb, as well as applications in which genetic engineering of a wide variety of cell types to express VHb have been used to enhance various processes of industrial importance. A specific practical application (using genetic engineering to enhance the production and alter the composition of the exopolysaccharides from *Ganoderma lucidum*, molecules with a variety of medical applications) is examined by Wang et al. Of particular interest, they show that the exopolysaccharides from the VHb-expressing cells have increased antioxidant activity. Finally, Wan et al. report on a novel use of the flavohemoglobin (Hmp) from *E. coli*. Flavohemoglobins are common microbial relatives of *Vitreoscilla* hemoglobin in which a flavin reductase domain is fused to the hemoglobin domain so that they can catalyze, by reduction and addition of oxygen, the conversion of nitric oxide to nitrate. The contribution by Wan et al., however, describes a different use, showing how fusion of all or part of *E. coli* Hmp to a variety of proteins increases their yield in engineered *E. coli*. Interestingly, the effect appears to be due to increased translation of the fusion proteins.

Taken together, the articles in this Special Issue trace the story of microbial hemoglobins from their discovery to the present day and point to future discoveries in which the various metabolic roles of microbial hemoglobins will be better defined and their use in practical applications will continue to broaden.

